# Development and clinical application of an automated machine learning-based delirium risk prediction model for emergency polytrauma patients

**DOI:** 10.3389/fphys.2025.1629329

**Published:** 2025-07-14

**Authors:** Zhenyi Liu, Yihao Huang, Long Li, Yisha Xu, Peng Wu, Zhigang Zhang, Tingyong Han, Liangjie Zhang, Ming Zhang

**Affiliations:** ^1^ Department of Emergency and Critical Care Medicine, The 945th Hospital of the Joint Logistics Support Force of the Chinese People’s Liberation Army, Ya’an, Sichuan, China; ^2^ Department of Psychosomatic Medicine, The 945th Hospital of the Joint Logistics Support Force of the Chinese People’s Liberation Army, Ya’an, Sichuan, China; ^3^ Emergency Department, Ya’an People’s Hospital, Ya’an, Sichuan, China; ^4^ Emergency Department, Yucheng District People’s Hospital of Ya’an, Ya’an, Sichuan, China; ^5^ Emergency Department, Mingshan District People’s Hospital of Ya’an, Ya’an, Sichuan, China; ^6^ Emergency Department, Affiliated Hospital of Ya’an Polytechnic College, Ya’an, Sichuan, China; ^7^ Emergency Department, Ya’an Hospital of Traditional Chinese Medicine, Ya’an, Sichuan, China

**Keywords:** delirium, polytrauma, machine learning, predictive model, explainable artificial intelligence

## Abstract

**Objective:**

To address the limitations of conventional delirium prediction models in emergency polytrauma care, this study developed an interpretable machine learning (ML) framework incorporating trauma-specific biomarkers and advanced optimization algorithms for risk stratification of delirium in emergency polytrauma patients.

**Methods:**

This multi-center retrospective observational cohort study was conducted across six hospitals in the Ya’an region. A total of 956 polytrauma patients admitted between January 2020 and December 2024 were enrolled, complying with the American Association for the Surgery of Trauma (AAST) diagnostic criteria for polytrauma. Demographic, clinical (e.g., Glasgow Coma Scale [GCS], Injury Severity Score [ISS]), and laboratory data (e.g., fibrin degradation products [FDP], lactate) were systematically collected. To address high-dimensional clinical heterogeneity, an Improved Flood Algorithm (IFLA)—enhanced with sine mapping initialization and Cauchy mutation perturbations—was integrated into an automated machine learning (AutoML) framework for simultaneous feature selection and hyperparameter optimization. Model performance was benchmarked against conventional algorithms (logistic regression [LR], support vector machine [SVM], extreme gradient boosting [XGBoost], LightGBM) using five-fold cross-validation. The SHapley Additive exPlanations (SHAP) framework quantified predictor contributions, and a MATLAB-based clinical decision support system (CDSS) was implemented for real-time risk stratification.

**Results:**

The improved algorithm significantly outperformed other algorithms on 12 standard test functions. The automated machine learning (AutoML) model achieved ROC-AUC and PR-AUC values of 0.9690 and 0.9611, respectively, on the training set, and 0.8929 and 0.8487, respectively, on the test set, both significantly higher than those of four other prediction models. The AutoML model identified 5 important features: Glasgow Coma Scale (GCS) score, lactate level, Clinical Frailty Scale (CFS), body mass index (BMI), and fibrin degradation products (FDP). The decision support system demonstrated clinical utility with net benefit across risk thresholds.

**Conclusion:**

This study provides a trauma-specific, interpretable ML tool that integrates GCS scoring and dynamic biomarker monitoring, enabling early delirium risk identification in emergency polytrauma. The framework demonstrates feasibility for integration into clinical workflows to improve trauma care quality.

## 1 Introduction

Trauma-related disorders have emerged as a critical global public health challenge. According to World Health Organization statistics, the socioeconomic burden attributable to traumatic injuries has risen to become the second leading contributor to the global disease burden (Galbraith et al., 2023). As a distinct subtype of trauma, polytrauma is characterized by complex pathophysiology, multi-system complications, and prolonged hospitalization, necessitating multidisciplinary collaborative care throughout treatment (Sumann et al., 2020). While damage control resuscitation (DCR) protocols significantly improve hemodynamic stability and survival rates, we observe increased neurological complication rates in this surviving cohort–particularly in patients requiring ≥6 units of blood transfusion. This complication profile reflects emergent pathophysiological perturbations in severely injured patients who survive initial resuscitation, rather than a direct consequence of DCR strategy (Kruithof et al., 2020). Among these, delirium—a severe neuropsychiatric syndrome with substantial prognostic implications—has been reported to affect 24% of emergency polytrauma patients. This condition not only prolongs mechanical ventilation duration and increases unplanned extubation risks but also induces long-term cognitive impairment, severely compromising patients’ quality of life (Von Rueden et al., 2017).

Although the American Guidelines for Critical Care Medicine explicitly recommend incorporating delirium screening into routine ICU care protocols, significant diagnostic gaps persist in clinical practice (Safdar et al., 2024). Studies indicate that healthcare providers actively identify delirium in 15%–20% of cases (Tonna et al., 2021). This disparity between knowledge and implementation may stem from three interrelated challenges: (1) the heterogeneous clinical manifestations driven by delirium’s complex pathophysiological mechanisms; (2) the high expertise requirements for administering validated assessment tools like the Confusion Assessment Method for the ICU (CAM-ICU); and (3) the inadequacy of traditional risk factor analysis in addressing dynamically evolving clinical features of polytrauma patients. Current delirium prediction models predominantly focus on geriatric or elective postoperative populations, with scarce systematic investigations into personalized model development for emergency trauma cohorts. This knowledge gap substantially hinders evidence-based implementation of precision preventive strategies (Heinrich et al., 2022).

Emerging evidence highlights the unique value of machine learning (ML) in predicting critical illness outcomes (Li et al., 2024; Fan et al., 2023; Tobin et al., 2024). Gong et al. developed a predictive model achieving an AUC of 0.845 (95% CI: 0.831–0.859), demonstrating the clinical potential of risk stratification in delirium management (Gong et al., 2023). However, significant challenges arise when adapting such models to emergency polytrauma scenarios. Key limitations include: (1) omission of trauma-specific indicators such as Injury Severity Score (ISS); (2) insufficient capacity of linear regression methods to capture complex variable interactions; and (3) unresolved conflicts between rapid decision-making demands and model usability in emergency settings (Rostam Niakan Kalhori, 2022). These shortcomings underscore the urgent need for context-specific predictive tools.

Building upon this rationale, our study innovatively integrates three pivotal components: (1) comprehensive trauma care cycle data collection; (2) adaptive ML algorithms optimized for dynamic clinical environments; and (3) implementation of the SHapley Additive exPlanations (SHAP) framework for transparent interpretation of model decisions. By synergizing advanced information technologies with traditional clinical research paradigms, this multidisciplinary approach aims to provide an intelligent solution for delirium prevention and management in emergency polytrauma patients, ultimately advancing the quality of trauma care delivery.

## 2 Methods

### 2.1 Study design

**FIGURE 1:**
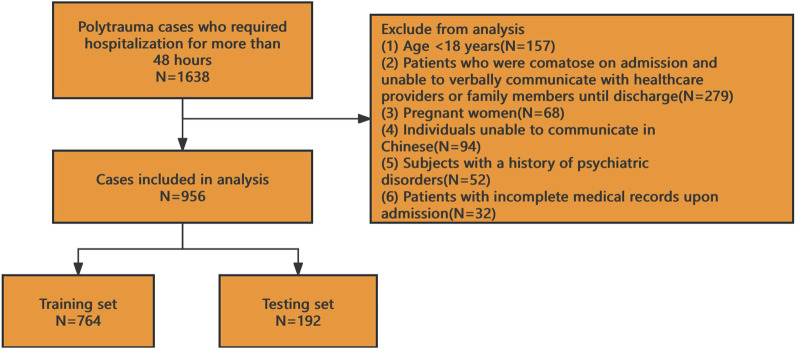
Patient selection flow chart.

This multicenter retrospective observational study was conducted across six hospitals in Ya’an, China. As a retrospective analysis, the requirement for informed consent was waived, and the study protocol received ethical approval from all participating institutions. We enrolled polytrauma patients admitted to these hospitals between January 2020 and December 2024. After applying inclusion and exclusion criteria, 956 patients were included in the final analysis (see Figure 1 for the patient selection flowchart).

Inclusion criteria: (1) Hospitalization for polytrauma meeting the diagnostic criteria of the American Association for the Surgery of Trauma (LaGrone et al., 2024); (2) ICU stay duration ≥48 h.

Exclusion criteria: (1) Age <18 years; (2) Patients who were comatose on admission and unable to verbally communicate with healthcare providers or family members until discharge; (3) Pregnant women; (4) Individuals unable to communicate in Chinese; (5) Subjects with a history of psychiatric disorders; (6) Patients with incomplete medical records upon admission.

### 2.2 Delirium diagnosis and data collection

All patient data were collected and assessed by psychosomatic physicians for delirium through medical record analysis and Confusion Assessment Method for the ICU (CAM-ICU) scoring conducted at 48 h post-ICU admission. This scoring method evaluates the following four criteria: (1) Acute onset or fluctuating mental status; (2) Inattention; (3) Altered level of consciousness; (4) Disorganized thinking. A diagnosis of delirium was confirmed if a patient met the first two criteria and one of the latter two criteria (Chen et al., 2021). All delirium diagnoses were established through retrospective chart review by two board-certified psychosomatic physicians following a standardized protocol. The primary physician conducted assessments using CAM-ICU criteria applied to medical records, with a secondary physician independently validating diagnoses across all identified cases to ensure inter-rater reliability.

Data collection: Patient data were retrieved via electronic medical record systems from multiple hospitals, consolidated, and uniformly processed by a single researcher. The dataset included: (1) Demographic information: Age, sex, height, weight, body mass index (BMI), Clinical Frailty Scale (CFS) (Shimura et al., 2017; Church et al., 2020), Charlson Comorbidity Index (CCI), smoking history, and alcohol use history; (2) Clinical parameters: Blood pressure, heart rate, body temperature, Glasgow Coma Scale (GCS) score, Revised Trauma Score (RTS), Injury Severity Score (ISS), and presence of traumatic brain injury (TBI); Traumatic brain injury (TBI) diagnosis was established through admission cranial CT scans interpreted by board-certified radiologists, with severity quantified using the Abbreviated Injury Scale (HEAD-AIS) specifically targeting neuroanatomical damage. Patients were classified as TBI-positive when HEAD-AIS ≥3 (moderate-to-severe injury), consistent with AAST/WSES organ injury grading standards. (3) Laboratory data: Fibrinogen, fibrin degradation products (FDP), hemoglobin, C-reactive protein (CRP), and lactate levels. All clinical parameters (including blood pressure, heart rate, GCS score, RTS, and ISS) were documented during the initial emergency department assessment immediately following patient admission. All laboratory biomarkers (fibrinogen, FDP, hemoglobin, CRP, lactate) were measured from venous blood samples collected at triage prior to any therapeutic interventions. TBI diagnosis was based on admission CT scans.

Missing data handling: The overall data completeness rate for the 956 included polytrauma patients was 97.43%. Missing rates varied across variables, with FDP exhibiting the highest missing rate (≤1% for other variables). Missing values were imputed using median replacement for continuous variables and mode substitution for categorical variables.

### 2.3 Model algorithm optimization and validation

To address the complexity of high-dimensional clinical data, we employed an automated machine learning (AutoML) model based on an optimization algorithm, which simultaneously performed feature selection and hyperparameter tuning. Traditional machine learning models were also included for performance comparison. All analyses were conducted in MATLAB 2024b. The Flood Algorithm (FLA) (Ghasemi et al., 2024), a novel swarm intelligence algorithm inspired by the complex movements of water masses, was used to optimize the AutoML framework. To enhance optimization performance, we improved the original FLA by integrating sine mapping initialization and Cauchy mutation perturbation strategies, resulting in the Improved Flood Algorithm (IFLA). The optimization capability of IFLA was validated using 12 standard benchmark functions from the IEEE CEC-2017 test suite (Sharma and Raju, 2024), including multimodal, hybrid, and composite functions such as Schwefel (F15), Rosenbrock (F6), and Lunacek Bi-Rastrigin (F23). Testing parameters: variable dimension = 10, population size = 30, maximum iterations = 500, with 30 independent runs for statistical robustness. Notably, these benchmark functions were used solely to evaluate the optimization performance of the swarm intelligence algorithm and did not participate in AutoML model training. The fitness function was defined as a direct mapping of the objective function value, with the optimization goal set to minimize the fitness value. Thus, a reduction in fitness value signifies improved algorithmic performance.

### 2.4 Model training and evaluation

To assess model quality in terms of performance, computational efficiency, interpretability, and robustness against underfitting/overfitting, we implemented five-fold cross-validation. The dataset was split into an 80% training set (for cross-validation) and a 20% test set. This approach effectively mitigated overfitting during training and improved prediction accuracy on the test set. We compared the performance of widely adopted and robust machine learning models, including logistic regression (LR), support vector machines (SVM), extreme gradient boosting (XGBoost), and LightGBM. These models were selected based on their proven performance and reliability in predictive analytics tasks. Quantitative evaluation metrics: Sensitivity (SEN), precision (PRE), specificity (SPE), accuracy (ACC), error rate (ER), and F1-score (F1). Primary comprehensive metrics: Area under the receiver operating characteristic curve (ROC-AUC) and precision-recall curve (PR-AUC). All metrics range from 0 to 1, with higher values indicating superior classification performance.

#### 2.4.1 Interpretability analysis

SHAP (SHapley Additive exPlanations) analysis, rooted in game-theoretic Shapley values, was employed to quantify feature contributions to model predictions. This method provides both global (model-wide) and local (individual sample-level) interpretability. Two types of SHAP visualizations were generated: (1) SHAP Summary Plot: Each point represents a feature’s SHAP value for a specific sample, color-mapped to reflect feature magnitude (blue: high values, white: low values), illustrating positive/negative relationships between features and predictions. (2) SHAP Importance Plot: Features are ranked by global importance based on absolute SHAP values, highlighting key predictors.

#### 2.4.2 Clinical decision system development

An interactive clinical decision support system was developed using MATLAB 2024a App Designer. This system integrates the prediction model, enabling clinicians to input clinical parameters via a structured interface and receive real-time predictions with therapeutic recommendations. The tool provides reliable and transparent decision-making assistance for clinical practice.

### 2.5 Statistical analysis

IBM SPSS v25.0 was used for conventional statistical analysis (significance: p < 0.05). Continuous variables were expressed as mean ± SD (normally distributed, Kolmogorov-Smirnov test) or median (IQR), and categorical variables as percentages.

## 3 Results

### 3.1 Baseline characteristics of study cohorts

The study included 956 patients, with 326 cases (34.1%) diagnosed with delirium. The dataset was randomly divided into a training set (80%, n = 764, delirium: 250 cases) and a test set (20%, n = 192, delirium: 76 cases). Baseline characteristics of both cohorts are summarized in Table 1.

**TABLE 1:** Baseline demographics and clinical characteristics of training and test sets.

Factor	Training set (n = 764)			Testing set (n = 192)		
	Delirium (n = 250)	No delirium (n = 514)	P value	Delirium (n = 76)	No delirium (n = 116)	P value
Age (year)	77.37 ± 8.42	58.28 ± 9.44	<0.001	77.81 ± 10.15	59.68 ± 8.49	<0.001
Sex, male (%)	149 (59.6)	304 (59.1)	0.904	47 (61.8)	73 (62.9)	0.879
BMI (kg/m^2^)	21.14 ± 4.32	22.6 ± 3.88	<0.001	21.18 ± 4.53	22.71 ± 4.04	<0.001
CFS (score)	4.58 ± 1.15	3.23 ± 0.68	<0.001	4.61 ± 1.21	3.28 ± 0.84	<0.001
Charlson index (score)	0 (0.2)	0 (0.1)	<0.001	1 (0.1)	0 (0.1)	0.022
Smoking (%)	43 (17.2)	101 (19.6)	0.417	14 (18.4)	23 (19.8)	0.809
Alcohol (%)	87 (34.8)	185 (35.6)	0.747	28 (36.8)	42 (36.2)	0.929
Systolic blood pressure (mmHg)	137.75 ± 23.16	140.85 ± 24.16	0.092	141.13 ± 25.05	142.31 ± 24.68	0.748
GCS score (score)	14 (13–15)	15 (14–15)	<0.001	14 (13–15)	15 (14–15)	<0.001
GCS score (score)	14.31 ± 2.52	15.85 ± 2.43	<0.001	14.38 ± 2.62	15.79 ± 2.51	<0.001
Heart rate (bpm)	84 (71,101)	82 (71.93)	0.168	82 (75,102)	81 (70.95)	0.427
Body temperature (°C)	36.3 (36, 36.8)	36.5 (36.1.36.9)	0.747	36.4 (36, 36.8)	36 (36.2, 37)	0.814
Hemoglobin (g/L)	121.83 ± 20.67	137.56 ± 17.89	<0.001	122.74 ± 19.52	138.15 ± 20.24	<0.001
Fibrinogen (mg/dL)	259.78 ± 42.17	257.88 ± 44.35	0.537	263.53 ± 51.05	259.76 ± 48.35	0.606
FDP (mg/L)	60.56 ± 18.79	16.74 ± 8.22	<0.001	62.74 ± 17.15	17.18 ± 6.85	<0.001
Lactate (mmol/L)	2.3 (1.5, 4)	2.1 (1.6, 2.9)	<0.001	2.5 (1.7, 3.4)	1.9 (1.4, 2.7)	<0.001
CRP (mg/L)	9 (3.40)	5 (2.14)	<0.001	8 (2.35)	5 (2.13)	<0.001
RTS (score)	7.83 (7.24–7.88)	7.86 (7.84–7.94)	<0.001	7.84 (7.34–7.86)	7.86 (7.84–7.94)	<0.001
ISS (score)	18 (10–27)	12 (9–19)	<0.001	18 (10–26)	12 (9–19)	<0.001
TBI(%)	117 (46.8)	215 (41.8)	0.193	34 (44.7)	46 (39.7)	0.485

### 3.2 Algorithm improvement performance evaluation

**FIGURE 2:**
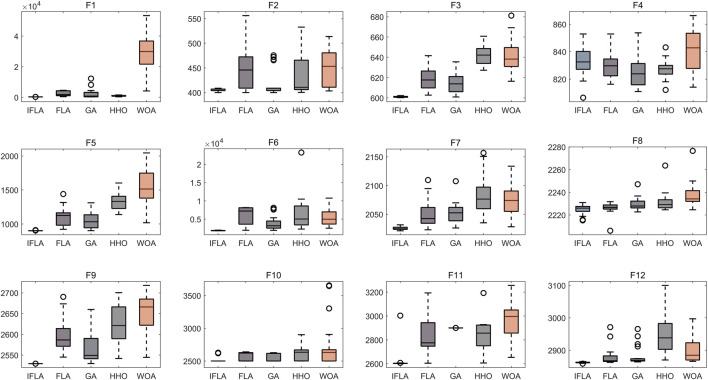
Comparison of swarm intelligence algorithm optimization performance.

**FIGURE 3:**
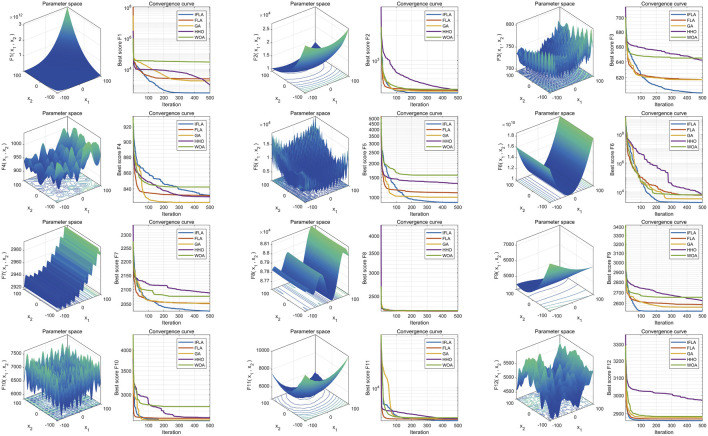
Comparison of convergence performance of swarm intelligence algorithms.

Based on 30 independent optimization runs, boxplots were generated to assess algorithm stability (Figure 2). The Improved Flood Algorithm (IFLA) demonstrated superior optimization stability compared to the original FLA and other benchmark algorithms across most test functions. Further convergence curve analysis (Figure 3) revealed that IFLA achieved faster convergence rates while maintaining the lowest risk of entrapment in local optima during iterations. These findings robustly validate IFLA’s enhanced global search capability and computational efficiency.

### 3.3 Model training performance

**FIGURE 4:**
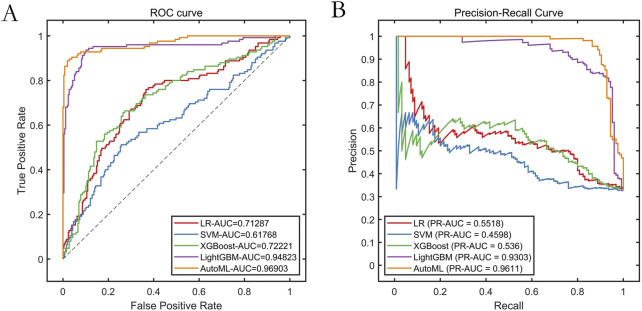
Training Set Performance Evaluation. Note: **(A)** ROC curve; **(B)** Precision-Recall curve.

The AutoML model exhibited optimal predictive performance on the training set: ROC-AUC: 0.9690; PR-AUC: 0.9611 (Table 2; Figure 4). Key features selected during model optimization included: Glasgow Coma Scale (GCS) score, lactate level, Clinical Frailty Scale (CFS), body mass index (BMI), and fibrin degradation products (FDP).

**TABLE 2:** Cross-validation performance metrics on the training set.

Models	PRE	SEN	SPE	ACC	F1	ROC-AUC	PR-AUC
LR	0.5781	0.2960	0.8949	0.6990	0.3915	0.7129	0.5518
SVM	—	0.0000	1.0000	0.6728	—	0.6177	0.4598
XGBoost	0.5929	0.5360	0.8210	0.7277	0.5630	0.7222	0.5360
LightGBM	0.9722	0.5600	0.9922	0.8508	0.7107	0.9482	0.9309
AutoML	0.8169	0.9280	0.8988	0.9084	0.8689	0.9690	0.9611

### 3.4 Test set validation

**FIGURE 5:**
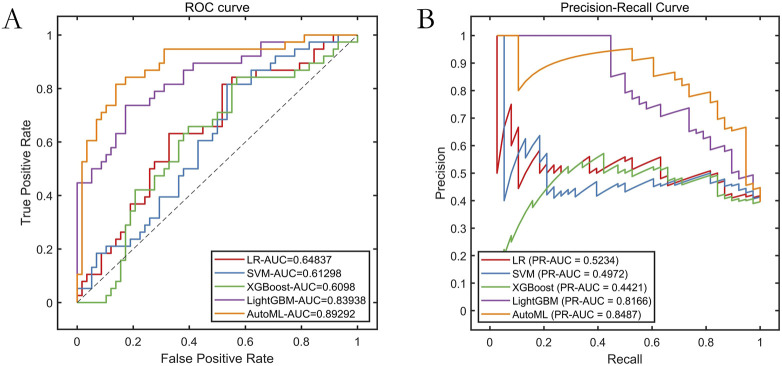
Testing Set Performance Evaluatione. Note: **(A)** ROC curve; **(B)** Precision-Recall curve.

The AutoML model maintained strong generalizability on the independent test set: ROC-AUC: 0.8929; PR-AUC: 0.8487 (Table 3; Figure 5).

**TABLE 3:** Predictive performance metrics on the testing set.

Models	PRE	SEN	SPE	ACC	F1	ROC-AUC	PR-AUC
LR	0.5294	0.2368	0.8621	0.6146	0.3273	0.6484	0.5234
SVM	—	0.0000	1.0000	0.6042	—	0.6130	0.4972
XGBoost	0.5333	0.4211	0.7586	0.6250	0.4706	0.6098	0.4421
LightGBM	0.7333	0.5789	0.8621	0.7500	0.6471	0.8394	0.8166
AutoML	0.9286	0.6421	0.9828	0.7292	0.7351	0.8929	0.8487

### 3.5 Interpretability analysis

**FIGURE 6:**
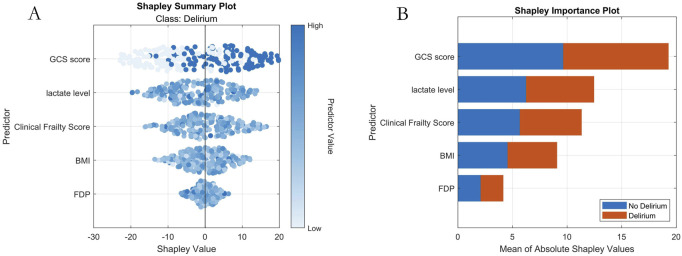
Machine Learning Interpretability Visualization. Note: **(A)** SHAP summary plot; **(B)** SHAP importance plot.

SHAP analysis quantified feature importance as follows (descending order): 1-GCS score; 2-Lactate level; 3-Clinical Frailty Scale; 4-BMI; 5-FDP (Figure 6).

### 3.6 Clinical utility

#### 3.6.1 Decision Curve Analysis

**FIGURE 7:**
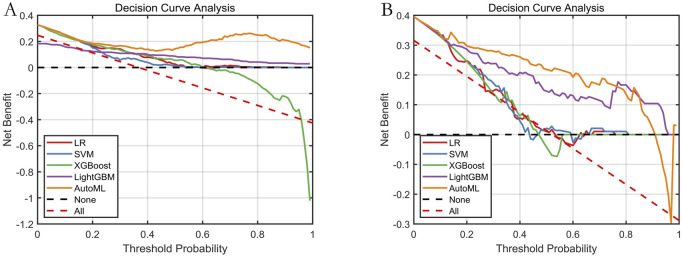
Decision Curve Analysis for Predictive Models. Note: **(A)** Training set; **(B)** Testing set. Net benefit (Y-axis) calculated against two extreme scenarios: “treat all” (red dashed) and “treat none” (black dashed).

The decision curve (Figure 7) demonstrated that applying the AutoML model to predict delirium risk provided greater clinical net benefit compared to alternative strategies across threshold probabilities.

#### 3.6.2 Decision support system

**FIGURE 8:**
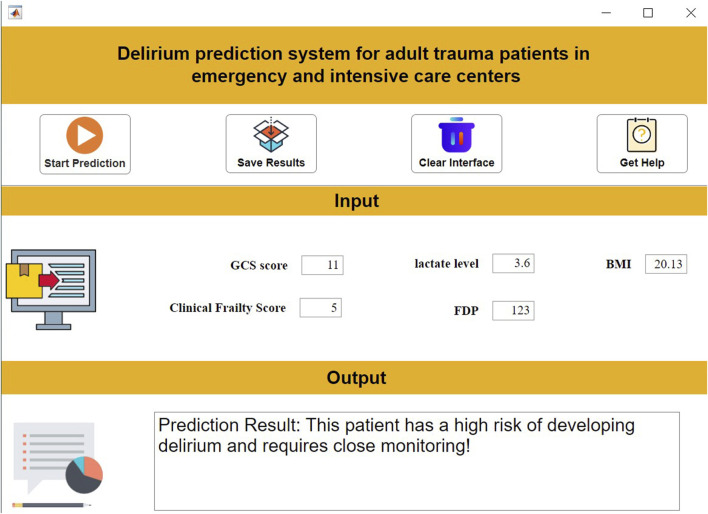
Clinical decision support system interface.

To address barriers in translating AI models to clinical practice (e.g., operational complexity), we developed an intuitive decision support system using MATLAB 2024a. The system allows clinicians to: Input patient features via a structured interface; Obtain real-time delirium risk predictions at the click of “Start Prediction”; Review evidence-based therapeutic recommendations. This tool significantly lowers implementation thresholds while ensuring interpretability and clinical relevance (Figure 8).

## 4 Discussion

Our study employed a multicenter retrospective design to develop an adaptive machine learning model based on an improved flood optimization algorithm (IFLA) for predicting delirium risk in emergency department (ED) patients with multiple trauma. Results demonstrated that the optimized IFLA model significantly outperformed traditional models (e.g., logistic regression and XGBoost) in key metrics including AUC and F1 scores. By integrating sine mapping initialization and Cauchy mutation perturbation strategies, the IFLA algorithm successfully overcame the local optimum trapping inherent in the conventional FLA, a finding corroborated by standard benchmark function tests. This innovative approach aligns with the algorithmic enhancements proposed by Gao et al. in COVID-19 prediction models (Gao et al., 2022). Through SHAP interpretability analysis, five critical predictors were identified: Glasgow Coma Scale (GCS) score, fibrin degradation products (FDP), lactate levels, body mass index (BMI), and Clinical Frailty Scale (CFS). Notably, GCS score exhibited the highest SHAP value contribution (26.8%). The real-time decision support system embedded in our model demonstrated favorable clinical acceptance during ED validation, indicating substantial translational potential.

Current delirium prediction research primarily focuses on medical or postoperative cohorts (Tobin et al., 2024; Gong et al., 2023; Rostam Niakan Kalhori, 2022; LaGrone et al., 2024; Chen et al., 2021; Shimura et al., 2017; Church et al., 2020; Ghasemi et al., 2024; Sharma and Raju, 2024; Gao et al., 2022; Liu et al., 2025; Shpakov et al., 2023; Saviano et al., 2023), with limited models specifically designed for trauma populations. Conventional linear regression approaches frequently exhibit inadequate predictive performance (AUC typically <0.85 (Matsuoka et al., 2021)) due to their limited capacity for modeling nonlinear relationships. Compared to rapid decision tree models developed in previous studies (Xie et al., 2022), our model demonstrated superior adaptability for polytrauma patients through the inclusion of trauma-specific indicators such as Injury Severity Score (ISS). While ISS significantly discriminated between groups, its utility as a reliable predictor was constrained by the limited critical trauma representation in our cohort, necessitating exclusion during model optimization. Future large-scale studies should validate its reintegration to enhance trauma-specific applicability. While Dana’s emergency informatics framework emphasizes data acquisition efficiency (Im et al., 2023), our study achieved concurrent feature selection and parameter optimization using AutoML technology, significantly enhancing computational efficiency. Importantly, previous research has largely overlooked the predictive value of coagulation markers (Stollings et al., 2021), whereas our findings highlight the critical role of FDP dynamics in delirium risk stratification, potentially mediated by neuroinflammatory cascades secondary to microcirculatory dysfunction in polytrauma (Bramley et al., 2021). Although Kang et al.'s sleep quality intervention reduced delirium incidence (Kang et al., 2023), its reliance on subjective clinician assessments contrasts with our objective predictive model that enables early targeted interventions.

Model refinement and SHAP analysis identified five core predictors, with their pathophysiological implications analyzed as follows: (1) GCS score: As a standardized consciousness assessment tool, GCS showed an inverse correlation with delirium risk. Severe brain injury (GCS ≤8) may trigger thalamocortical feedback loop dysregulation (attentional deficits), locus coeruleus norepinephrine system hyperactivation (neurotransmitter imbalance), and blood-brain barrier disruption (neuroinflammation via IL-6/TNF-α infiltration) (Raquer et al., 2024). For ED physicians, dynamic GCS monitoring (particularly in TBI patients) facilitates early identification of high-risk individuals (GCS ≤12), enabling timely preventive measures. (2) Lactate levels: This biomarker of tissue hypoperfusion quantifies oxygen metabolism dysregulation. Levels >2 mmol/L promote delirium via three pathways: 1) astrocytic glutamate uptake inhibition (excitotoxicity); 2) microglial TLR4/NF-κB pathway activation (neuroinflammation); 3) cerebral acidosis impairing neurotransmitter dynamics (Qian et al., 2024). The sharp SHAP value increase at >4 mmol/L suggests a threshold effect. Integrating central venous oxygen saturation monitoring for fluid resuscitation optimization (as shown by Taylor et al. (Taylor et al., 2022)) could reduce delirium incidence by 19%. (3) Clinical Frailty Scale (CFS): Scores ≥5 indicate depleted physiological reserves, amplifying trauma effects through immunosenescence (sustained inflammation), autonomic dysregulation (circadian disruption), and altered pharmacokinetics (sedative accumulation) (Zhang et al., 2021; Mazzola et al., 2021). Our model ranks CFS third in SHAP importance, warranting “precision trauma care” strategies including nutritional support (protein ≥1.2 g/kg/day), early mobilization (bedside sitting within 24h), and benzodiazepine restriction. (4) BMI: The U-shaped delirium risk (optimal range 18.5–24.9 kg/m^2^ (Feinkohl et al., 2023)) reflects dual mechanisms: low BMI exacerbates catabolism (neurotransmitter precursor deficiency), while obesity induces leptin resistance (insulin resistance/BBB disruption). Obese patients require vigilance for occult hypoperfusion from intra-abdominal hypertension, whereas underweight patients may benefit from enteral nutrition with branched-chain amino acids (Fu et al., 2024). (5) FDP: Elevated FDP (>20 μg/mL) signals coagulopathy via complement C5a activation (microvascular NETosis) and competitive fibrinogen inhibition (hemorrhagic risk) (Payne et al., 2024). Six-hourly FDP monitoring combined with tranexamic acid administration may mitigate microcirculatory dysfunction-related delirium (Lu et al., 2024).

Despite constructing this automated prediction model, limitations persist in data quality and clinical implementation: Data source bias: Though standardized across six regional hospitals, geographical disparities in trauma protocols and monitoring standardization may introduce bias. While multicenter recruitment enhances external validity, the moderate cohort size limited subgroup analyses for rare trauma phenotypes. We also recognize inherent inter-hospital variability in scoring systems despite standardized training. Future large-scale validation should prioritize algorithmic adaptation to institution-specific documentation patterns using federated learning frameworks. While median imputation mitigates leakage risk, FDP remains susceptible to bias due to its higher missing rate (2.6%). Future studies should employ advanced methods like multiple imputation chained equations (MICE) for variables exceeding 2% missingness. Retrospective acquisition of FDP values, though demonstrating critical prognostic value, necessitates future validation of our dynamic prediction updating protocol in prospective studies employing point-of-care viscoelastic testing to eliminate turnaround delays. Model constraints: Missing core variables and incomplete capture of nonlinear interactions may reduce sensitivity in complex trauma scenarios. Temporal resolution: Static-input prediction systems face intrinsic latency in dynamic ED environments requiring real-time biomarker feedback (e.g., rapidly changing lactate/FDP). Study design limitations: While retrospective validation provides preliminary evidence, prospective cohorts remain essential for examining delirium’s temporal progression and intervention dynamics.

The Intensive Care Big Data Steward Consensus publishes future industry standards in this area (Su et al., 2024), this consensus makes 29 recommendations on the following five parts: Concept of intensive care big data, Important scientific issues, Standards and principles of database, Methodology in solving big data problems, Clinical application and safety consideration of intensive care big data. Aligned with the Intensive Care Big Data Consensus, our future research framework will embed its 29 evidence-based recommendations across five core dimensions: establishing harmonized multimodal trauma databases adhering to standardized ICU data protocols, implementing federated learning architectures for privacy-preserving multicenter integration, applying advanced AutoML optimization for feature engineering, developing clinical translation pathways within evidence-based safety parameters, and creating real-time SHAP interpretability dashboards for predictive governance. This structured methodology will operationalize the consensus guidelines—particularly regarding scientific question formulation, database standardization, and ethical computational methods—as applied to dynamic delirium prediction in trauma ecosystems. It includes the following aspects: (1) Data integration: Establish multimodal trauma databases incorporating real-time vital signs, continuous EEG, and cytokine profiles to transcend retrospective “time-slice” limitations. Our real-time data pipeline implements sliding-window RNNs for hourly risk-score updates coupled with automatic quarterly calibration audits against AAST/WSES standards, ensuring temporal relevance through federated learning with patient-level partitioning. (2) Algorithm enhancement: Develop spatiotemporal architectures (e.g., temporal convolutional networks for biomarker trends, graph neural networks for multi-organ injury topology) to transition from “point prediction” to “process warning.” We implement federated learning and ensemble transition strategies where legacy models are progressively weighted with PAN-GAN-synthesized newer cohorts, enabling continuous adaptation to clinical practice shifts during model development. (3) Clinical translation: Implement edge computing-embedded decision systems integrated with bedside monitors/laboratory streams during the “golden hour” of trauma care. Edge-computing-embedded decision systems integrated with bedside monitors/laboratory streams during the “golden hour” now incorporate SHAP-based performance dashboards triggering alerts for critical predictor drift (e.g., >1.5σ change in GCS or FDP contributions). Future iterations should integrate multimodal neurological assessments such as the Full Outline of UnResponsiveness (FOUR) scale to enhance sensitivity in patients with communication barriers (e.g., intubation, aphasia). To advance translational implementation, our research road now explicitly prioritizes EHR interoperability through three parallel initiatives: Development of HL7 FHIR-compliant APIs enabling automated data exchange with hospital information systems at participating centers; Design of clinician-centered mobile interfaces with offline functionality to support bedside risk stratification during resuscitation, featuring real-time SHAP visualizations when FDP trends exceed >1.5σ baseline deviations; Prospective workflow integration trials launching Q4-2026 to quantify adoption metrics and time-motion efficiency gains using the System Usability Scale across three trauma networks. This aligns with our prioritization of spatiotemporal feature engineering and edge-computing integration, potentially improving real-time risk stratification during the “golden hour” of trauma care. Synergizing evidence-based medicine with AI could enable personalized interventions (e.g., circadian modulation for high-CFS patients, anticoagulant optimization for coagulopathic cases), ultimately creating a closed-loop “prediction-intervention-verification” ecosystem through SHAP-guided precision pathways.

## Data Availability

The raw data supporting the conclusions of this article will be made available by the authors, without undue reservation.
